# Endocrine Disorders and Genital Infections Impair Gynecological Health in APECED (APS-1)

**DOI:** 10.3389/fendo.2021.784195

**Published:** 2021-11-30

**Authors:** Viivi Saari, Saila Laakso, Aila Tiitinen, Outi Mäkitie, Elina Holopainen

**Affiliations:** ^1^ Children’s Hospital and Pediatric Research Center, University of Helsinki and Helsinki University Hospital, Helsinki, Finland; ^2^ Research Program for Clinical and Molecular Metabolism, Faculty of Medicine, University of Helsinki, Helsinki, Finland; ^3^ Folkhälsan Research Center, Folkhälsan Institute of Genetics, Helsinki, Finland; ^4^ Department of Obstetrics and Gynecology, University of Helsinki and Helsinki University Hospital, Helsinki, Finland; ^5^ Department of Molecular Medicine and Surgery, Karolinska Institutet and Clinical Genetics, Karolinska University Hospital, Stockholm, Sweden

**Keywords:** endocrine, autoimmunity, female, premature ovarian insufficiency, ovary, androgen, APS-1

## Abstract

**Objective:**

In autoimmune polyendocrinopathy-candidiasis-ectodermal dystrophy (APECED) defects in the autoimmune regulator gene lead to impaired immunotolerance. We explored the effects of immunodeficiency and endocrinopathies on gynecologic health in patients with APECED.

**Design:**

Cross-sectional cohort study combined with longitudinal follow-up data.

**Methods:**

We carried out a gynecologic evaluation, pelvic ultrasound, and laboratory and microbiologic assessment in 19 women with APECED. Retrospective data were collected from previous study visits and hospital records.

**Results:**

The study subjects’ median age was 42.6 years (range, 16.7-65.5). Sixteen patients (84%) had premature ovarian insufficiency, diagnosed at the median age of 16.5 years; 75% of them used currently either combined contraception or hormonal replacement therapy. In 76% of women, the morphology and size of the uterus were determined normal for age, menopausal status, and current hormonal therapy. Fifteen patients (79%) had primary adrenal insufficiency; three of them used dehydroepiandrosterone substitution. All androgen concentrations were under the detection limit in 11 patients (58%). Genital infections were detected in nine patients (47%); most of them were asymptomatic. Gynecologic *C. albicans* infection was detected in four patients (21%); one of the strains was resistant to azoles. Five patients (26%) had human papillomavirus infection, three of which were high-risk subtypes. Cervical cell atypia was detected in one patient. No correlation between genital infections and anti-cytokine autoantibodies was found.

**Conclusions:**

Ovarian and adrenal insufficiencies manifested with very low androgen levels in over half of the patients. Asymptomatic genital infections, but not cervical cell atypia, were common in female patients with APECED.

## Introduction

Autoimmune polyendocrinopathy-candidiasis-ectodermal dystrophy (APECED), also called autoimmune polyendocrine syndrome type I (APS-I), is a rare disorder arising from mutations in the autoimmune regulator (*AIRE*) gene (1). Mutations in *AIRE* cause disturbances in T cell maturation and development of central tolerance in the thymus, leading to autoimmune manifestations (2). The classic triad of APECED is characterized by hypoparathyroidism (HP), primary adrenocortical insufficiency (PAI), and chronic mucocutaneous candidiasis (CMC). Other potential disease manifestations include e.g. premature ovarian insufficiency (POI), hypothyroidism, malabsorption, and autoimmune hepatitis (3).

CMC is the most common and often one of the first manifestations in APECED (4). CMC typically manifests at oral mucosa, but may also affect the mucosa of the esophagus, intestine, or genital tract (5). Patients need preventive, long-term, or intermittent antifungal medications, and consequently *Candida albicans* strains may develop resistance to azoles (6). Studies in APECED have found oral CMC to correlate with circulating antibodies against Th17 related cytokines IL-22 and IL-17F (7, 8). These anti-cytokine autoantibodies are common in patients with APECED even before any clinical disease manifestations develop (5, 9, 10). However, the effects of anti-cytokine autoantibodies remain largely unknown. Oral CMC can predispose to oral squamous cell carcinoma (11), but it is unknown if CMC may also predispose to cell atypia on other mucosal surfaces, such as genital mucosa. Furthermore, it is not known whether patients with APECED have increased risk for other gynecologic infections including human papillomavirus (HPV) infections and consequently for cervical neoplasms.

In addition to vulnerability to infections, also certain endocrinopathies including POI and PAI, have potential effects on gynecologic health in female patients with APECED (12). In Finnish female patients with APECED, POI is the third most common endocrinopathy, manifesting in 70% at the median age of 16 years (12). In other APECED cohorts, the prevalence of POI varies from 9% to 71% (5, 13–15). POI leads to hypoestrogenism and lack of ovarian androgens (16). PAI is found in 22-83% of patients with APECED resulting in lack of adrenal androgens (5, 13–15). Long-term hormone replacement therapy (HRT) is needed to prevent consequences of hypoestrogenism (17). However, studies on adrenal androgen substitution have been controversial (18, 19). To our knowledge, no study has evaluated the effects of POI and PAI on gynecologic health in patients with APECED.

In this study with cross-sectional evaluation combined with longitudinal follow-up data, we describe in detail the patients’ gynecologic health including hormonal state, pelvic ultrasound, cell and microbe samples of cervix and vagina, and presence of autoantibodies against cytokines. We aimed to clarify the effects of immunodeficiency and endocrinopathies on gynecologic health in APECED in order to guide patient management.

## Materials and Methods

### Patients

Patients were identified from the large Finnish cohort of over 90 patients with APECED (4, 20). All living female patients aged 11 years and older *(n=*33) were invited to participate in the study, 20 (61%) of them consented. One patient was excluded from final analysis due to prepubertal status based on clinical assessment and laboratory parameters. Eleven patients (11/19, 58%) had also participated in an earlier gynecologic study in 1995, allowing longitudinal data evaluation. Informed written consent was obtained from study participants or their guardians (subjects <18 years). Ethical approval was obtained from the Research Ethics Committee of the Hospital District of Helsinki and Uusimaa.

### Clinical Assessments

Patients were interviewed for detailed medical history and measured for height (to the nearest 0.1 cm) and weight (in light clothing to the nearest 0.1 kg). Patients underwent a complete gynecologic examination and pelvic ultrasound (GE Healthcare Voluson S6), performed by gynecologist (EH). Clinical estrogenization of external genitalia and vagina were estimated. Pubertal status was determined according to Tanner’s scale for breast development and pubic hair (21). Morphology of the uterus and ovaries was assessed by ultrasound and compared with age and menopausal status-based reference values (22). Normal size of the uterus was considered as a sign of adequate estrogenization. The maximum anterior-posterior distance measured in the mid-portion of the uterine body on a sagittal view, and median length of the uterine corpus, measured from the fundus to the internal orifice of the uterus on a sagittal view, were determined (23, 24). Antral follicle count was determined when technically possible and considered normal if it was between 10^th^ and 90^th^ percentiles of age-specific normal values (25).

Hospital records were reviewed for pubertal development, development of POI, fertility, previous gynecologic examinations, and gynecologic surgical operations. POI was diagnosed if the patient had not reached stage 2 of breast development by age 13 years or menarche by 15 years or she had POI symptoms with secondary amenorrhea for 4 months or, in the case with primary or secondary amenorrhea, if follicle-stimulating hormone (FSH) was over 40 IU/L before the age of 40 years (16, 26). APECED manifestations (CMC, HP, PAI, diabetes, growth hormone deficiency, hypothyroidism, alopecia, asplenia, constipation, diarrhea, enamel hypoplasia, gastritis, hepatitis, keratoconjunctivitis, nephritis, oral squamous carcinoma, rash with fever, vitiligo) and their age of onset, were reviewed.

### Hormone and Autoantibody Assays

Blood samples were obtained without fasting. Serum concentrations of Anti-Müllerian hormone (AMH), FSH, luteinizing hormone (LH), estradiol, testosterone, androstenedione, and dehydroepiandrosterone (DHEAS) were measured and interpreted regarding phase of cycle or use of hormonal therapy.

AMH was quantitated with an electrochemiluminometric assay on a cobase 411 automatic immunoanalyzer (Elecsys AMH Plus, Roche Diagnostics). Limit of detection (LoD) was 0.01 µg/L and limit of quantitation (LoQ) 0.03 µg/L. Intra-assay coefficient of variations (CV%) was < 2% and inter-assay CV% <5% in the range 0.2–19 µg/L of AMH. Age-specific limits of AMH were applied (27). LH and FSH concentrations were measured using an electrochemiluminescence immunoassay (Abbott Diagnostics). Method for LH had a LoQ of 0.07 IU/L; intra-assay CV% was 3% and inter-assay CV% 7%. Method for FSH had a LoQ of 0.3 IU/L; intra-assay CV% was 7% and inter-assay CV% 7%.

Serum estradiol, androstenedione, and testosterone were extracted with liquid-liquid extraction using diethyl ether. Androstenedione sample extracts were analyzed on a liquid chromatography-tandem mass spectrometry system (LC-MS/MS) comprising a TQ5500 triple quadrupole mass spectrometer (AB Sciex) and an Agilent Technologies (Santa Clara) series 1200 HPLC system with a binary pump. The LC-MS/MS method for androstenedione had a LoQ of 0.1 nmol/L; inter-assay CV% at 3.2, 8.0, and 19 nmol/L was 5.5%, 6.4%, and 8.1%, respectively. Testosterone sample extracts were analyzed on an LC-MS/MS system comprising an API3000 triple quadrupole mass spectrometer (AB Sciex) and an Agilent Technologies (Santa Clara) series 1100 HPLC system with a binary pump. The LC-MS/MS method for testosterone had a LoQ of 0.2 nmol/L; inter-assay CV% at 4.7, 15.8, and 25.1 nmol/L was 5.2%, 4.6%, and 5.3%, respectively. Estradiol was quantitated by LC-MS/MS using a TQ5500 mass spectrometer (AB Sciex). The between-run CV% were 4.5-5.7% for serum samples at concentrations of 317-898 pmol/L and the detection limit was 0.01 nmol/L (28). DHEAS sample extracts were measured using a direct chemiluminescent immunoassay (Siemens Atellica OM 1600 Analyzer). The detection limit was 0.08 µmol/L; intra-assay CV% was 7%, and inter-assay CV% 7%.

Autoantibodies against interferon-α (IFN-α), interferon-γ (IFN-γ), interleukin-17A (IL-17A), interleukin-17F (IL-17F), and interleukin-22 (IL-22) were detected by ELISA and by flow cytometric bead array (Luminex analyzer) in an accredited laboratory as previously described (7, 29).

### Cellular and Microbe Samples

*Candida* culture of vulva and vagina, cervical HPV tests, and PAP smears were obtained. *C. albicans* swabs were cultured in CHROM-agar dish at 37°C for one week. Growth was identified with the bioMerieux VITEK-MS Maldi-tof method. Resistance of *C. albicans* to medications was tested with Etest (bioMerieux). If the *C. albicans* population was resistant to fluconazole, resistance to other fungal medication (voriconazole, amphotericin B, and micafungin) was tested. In Etest, *C. albicans* was mixed with 0.9% NaCl to cultivate a mixture of 1.2 Macfarland. The mixture was spread to RPMI-dish, where E-test strips were added. MIC-value was assessed on two consecutive days.

Cervical samples for HPV tests were taken by APTIMA cervical collection and transport KIT. HPV subtypes were analyzed by PCR and Luminex suspension array technology (30). The test detects 15 high-risk HPV types, six potentially high-risk types, and 19 low-risk HPV types. Pap smears were analyzed using standard clinical protocols.

### Statistical Analysis

Median (range) was used to report the results. Mann-Whitney U or Spearman correlation test was used for continuous variables and Fisher’s exact test for categorical variables. A *P* value under 0.05 was considered statistically significant. IBM SPSS statistics version 25 was used for statistical analysis.

## Results

### Patient Characteristics and Gynecologic History

The median age of the 19 study participants was 42.6 years (range, 16.7–65.5). All patients had biallelic *AIRE* mutations. Fifteen patients (79%) were homozygous for the Finnish major mutation c.769C>T (p.Arg257Ter) in the *AIRE*. Three patients had the major mutation compounded with c.932G>A (p.Cys311Tyr) *(n=*1), c.967_979del13 (p.Leu323fs) *(n=*1), or c.137C>G (p.Thr46Arg) *(n=*1). One patient had c.901G>A (p.Val301Met) and an intronic variant c.996-17G>A in the *AIRE*.

The cohort characteristics are presented in **Tables 1**, **2**. Fourteen patients (74%) had gone through spontaneous pubertal development. In five patients (26%), HRT had been used to induce or to complete pubertal development and to induce menarche. Altogether 16 patients (84%) had developed POI at the median age of 16.5 years (11.3–36.5). Ten patients (63%) with POI currently used HRT, and two used combined contraception (**Table 2**). At the time of evaluation, five women were considered postmenopausal (**Table 1**). PAI was diagnosed in 15 patients (79%) in the median age of 10.8 years (2.5–32.9), and three of them (20%) used dehydroepiandrosterone (DHEA) substitution. None of the patients currently used testosterone therapy. Three patients (16%) had used fluoxymesterone to induce pubic hair growth from the median age of 11.4. Only one patient had used testosterone substitution as an adult. Eight patients (42%) had been pregnant (**Table 1**).

**Table 1:** History of pubertal development, manifestations of APECED and pregnancies in the 19 patients with APECED.

Characteristic	Median/*n*	Range/%
Age at study visit (years)	42.6	16.7 – 65.5
Age at stage 2 of breast development	11.9	10.6 – 17.7
Age at stage 2 of pubic hair growth	13.1	10.6 – 16.7
Age at menarche		
Spontaneous *(n=*14)	13.8	11.0 – 15.5
Medically induced *(n=*5)	17.7	14.3 – 18.5
Age at POI*^a^ (n=*16)	16.5	11.3 – 36.5
Age at menopause/discontinuation of HRT		
Physiological menopause *(n=*1)	48.3	–
Age at the discontinuation of HRT *(n=*4)	51.4	47.3 – 53.5
Number of APECED manifestations	8	4 – 11
POI	16	84%
PAI	15	79%
HP	19	100%
CMC	18	95%
Ever been pregnant	8	42%
Number of pregnancies	10	
Spontaneous	5	
Ovum donation	5	

POI, premature ovarian insufficiency; PAI, primary adrenocortical insufficiency; HP, hypoparathyroidism; CMC, chronic mucocutaneous candidiasis.

^a^Diagnostic criterion: delayed puberty (patient has not reached stage 2 of breast development by the age of 13 years or menarche by the age of 15 years) or POI symptoms with amenorrhea and/or FSH over 40 IU/L under the age of 40 years.

**Table 2:** Patient characteristics, state of hormonal replacement therapy, and ultra-sound measurements at the time of appointment in the 19 patients with APECED.

Variable	Median/*n*	Range/%
Height (cm)	161.0	151.2 – 174.0
Height SDS	-1.1	-2.9 – + 1.3
Weight (kg)	62.0	34.7 – 100.0
BMI (kg/m^2^)	23.2	13.8 – 42.7
Underweight (< 18.5)	4	21%
Normal weight (18.5–25)	10	53%
Overweight (> 25)	5	26%
Spontaneous menstrual cycle at study visit	2	11%
Current hormonal therapy in use	12	63%
Combined contraception	2	11%
Systemic HRT *^a^*	10	53%
Transdermal estrogen + oral progesterone	1	10%
Oral estrogen + oral progesterone	6	60%
Oral estrogen + levonorgestrel releasing intrauterine device	1	10%
Oral estrogen without progesterone	2	20%
Vaginal estrogen only	1	8%
Normal size of the of uterus *^b^ (n=*17) *^c^*	13	76%
Abnormal size of the uterus *^b^ (n=*17) *^c^*	4	24%
AP of uterus corpus (mm) (*n=*17*)*	32.0	15.0 – 49.0
Length of uterus corpus (mm) (*n=*15)	39.0	30.0 – 59.0
Ovaries identifiable (*n=18) ^d^*	12	67%
Ovarian AFC in a normal range	2	17%

HRT, hormone replacement therapy; AP, anterior posterior; AFC, antral follicle count.

^a^systemic HRT and vaginal estrogen, n=3 (16%); ^b^assessed in relation to patient’s age, phase of menstrual cycle, menopausal status, and HRT (21); ^c^uterus surgically removed from 2 patients; ^d^ovaries surgically removed from 1 patient, ovaries identifiable in 12 patients.

Three patients (16%) had undergone gynecological surgery; abdominal hysterectomy with bilateral salpingo-oophorectomy *(n=*1), hysterectomy *(n=*1), and uterine artery embolization *(n=*1). Indications were excessive gynecological bleeding, miscarriage, and uterine leiomyomas, respectively.

### Gynecologic Status

At clinical evaluation, all patients had completed pubertal development. Their median height was 161.0 cm (151.2-174.0 cm; **Table 2**). All had normal breast development. Pubic hair was absent in seven patients (37%): all of them had PAI and had previously had at least Tanner stage 2 for pubic hair growth and none of them currently used DHEA substitution.

External genitalia and vaginal epithelium were considered normal in 11 patients (58%). Vulvovaginal atrophy was found in five patients (26%). Yeast infection and unspecific vaginitis was clinically suspected in three and one patient, respectively.

Pelvic ultrasound was performed vaginally in 18 patients and abdominally in one patient (**Table 2**). In 13 (13/17, 76%) women, the morphology and size of the uterus were determined normal for age, stage of menstrual cycle, menopausal status, and current hormonal therapy. The four patients with uterus size below reference values were currently using HRT. No ovarian pathology was diagnosed in 12 patients with identifiable ovaries. Consistently with POI, antral follicle count was diminished in most patients (10/12, 83%).

### Hormonal Levels

**Figure 1:**
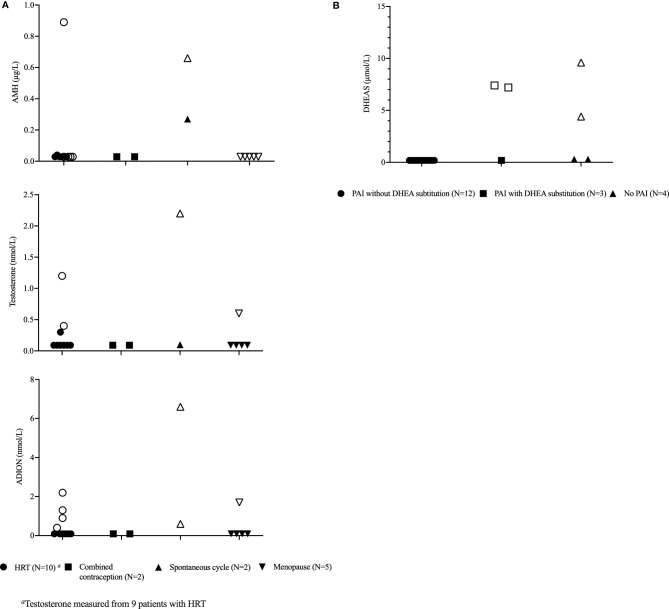
Ovarian and adrenocortical hormones in 19 female patients with APECED. **(A)** Anti-Müllerian hormone (AMH), androstenedione, and testosterone levels in relation to ovarian function and hormonal replacement therapy (HRT). **(B)** Dehydroepiandrosteronesulfate (DHEAS) levels in relation to the presence of primary adrenocortical insufficiency (PAI) and use of dehydroepiandrosterone (DHEA) substitution. Values in transparent markings are considered to be within the normal range.

FSH, LH, and estradiol levels were consistent with the cycle phase or hormonal treatment (data not shown). AMH was below the detection limit in 15/19 patients, all with POI or menopausal (**Figure 1A**). AMH was subnormal in one female (0.27 µg/L) who still had spontaneous cycles reflecting the depletion of ovarian function. Similarly, AMH was subnormal (0.04 and 0.89 µg/L) in two patients with POI and regular HRT substitution.

Androgen levels were low in patients with APECED (**Figures 1A, B**). Testosterone, androstenedione, and DHEAS were below the detection limit in 67%, 63%, and 68% of the patients, respectively. DHEAS was below the detection limit or low normal in all patients with PAI who did not have DHEA substitution (**Figure 1B**). All androgens were below the detection limit in 11 patients (58%).

### Genital Infections and Cervical Cell Atypia

History of genital infections and results of the previous and current gynecologic microbial and Pap smear samples are presented in **Table 3**. Only two patients did not report any previous genital infections. Seventeen patients (89%) had experienced at least one previous genital *C. albicans* infection. Six (35%) of them reported recurrent genital *C. albicans*.

**Table 3:** Previous and current gynecological infections and pap smear findings in 19 patients with APECED.

Variable	*n*	%
Previous gynecological infections		
*C. albicans* infections	17	89%
Bacterial vaginosis	5	26%
Condyloma	3	16%
Chlamydia	1	5%
History of abnormal pap smears *^a^*	7	37%
Gynecological visits in 1995 (11/19) *^b^*		
Oral anti-fungal medication *^c^*		
No	8	73%
Therapeutic dose	3	27%
Prophylactic dose	–	–
Genital *C. albicans* infection	3	27%
Bacterial vaginosis	2	18%
Cell atypia in pap smear	1	9%
Current infections and gynecological findings *^c^*		
Oral anti-fungal medication *^d^*		
No	15	79%
Therapeutic dose	3	16%
Prophylactic dose	1	5%
Genital *C. albicans* infection	4	21%
Only vulva	2	11%
Vulva and vagina	2	11%
HPV test (19/19)		
Positive	5	26%
High-risk subtype	3	60%
Pap smear (19/19)		
Bacterial vaginosis	2	11%
No cell atypia	18	95%
Cell atypia	1	5%

^a^Cell atypia ASCUS or more/class 2 or higher; ^b^11 patients participated in a gynecologic study also in 1995; ^c^From the gynecological visits in 2019-20; ^d^Only systemic anti-fungal medication was used.

The proportion of patients with CMC, bacterial vaginosis, or cell atypia was similar in the two study visits approximately 25 years apart. Currently, four patients (21%) had *C. albicans* growth either in vulva or vagina. In one of them, *C. albicans* was resistant to fluconazole and voriconazole but sensitive to amphotericin B and micafungin. The patient was using prophylactic oral fluconazole. Three other *C. albicans* strains were sensitive to fluconazole. Three of the four patients with *C. albicans* had abnormal findings in gynecological examination; one patient had clinical suspicion of candidiasis, one had hypoestrogenic vulvar mucosa, and one had clinical vulvovaginitis. None of them had cell atypia in the pap smear.

Two patients (11%) had been vaccinated against HPV. HPV was found in five patients (26%); three (16%) had high-risk subtypes 31, 33, 52 or 68; one of them had three of these subtypes. Cell atypia (low-grade squamous intraepithelial lesion) in Pap smear was detected in one patient whose HPV test remained negative. Patients with bacterial vaginosis were clinically asymptomatic.

### Autoantibodies to Cytokines

**Figure 2:**
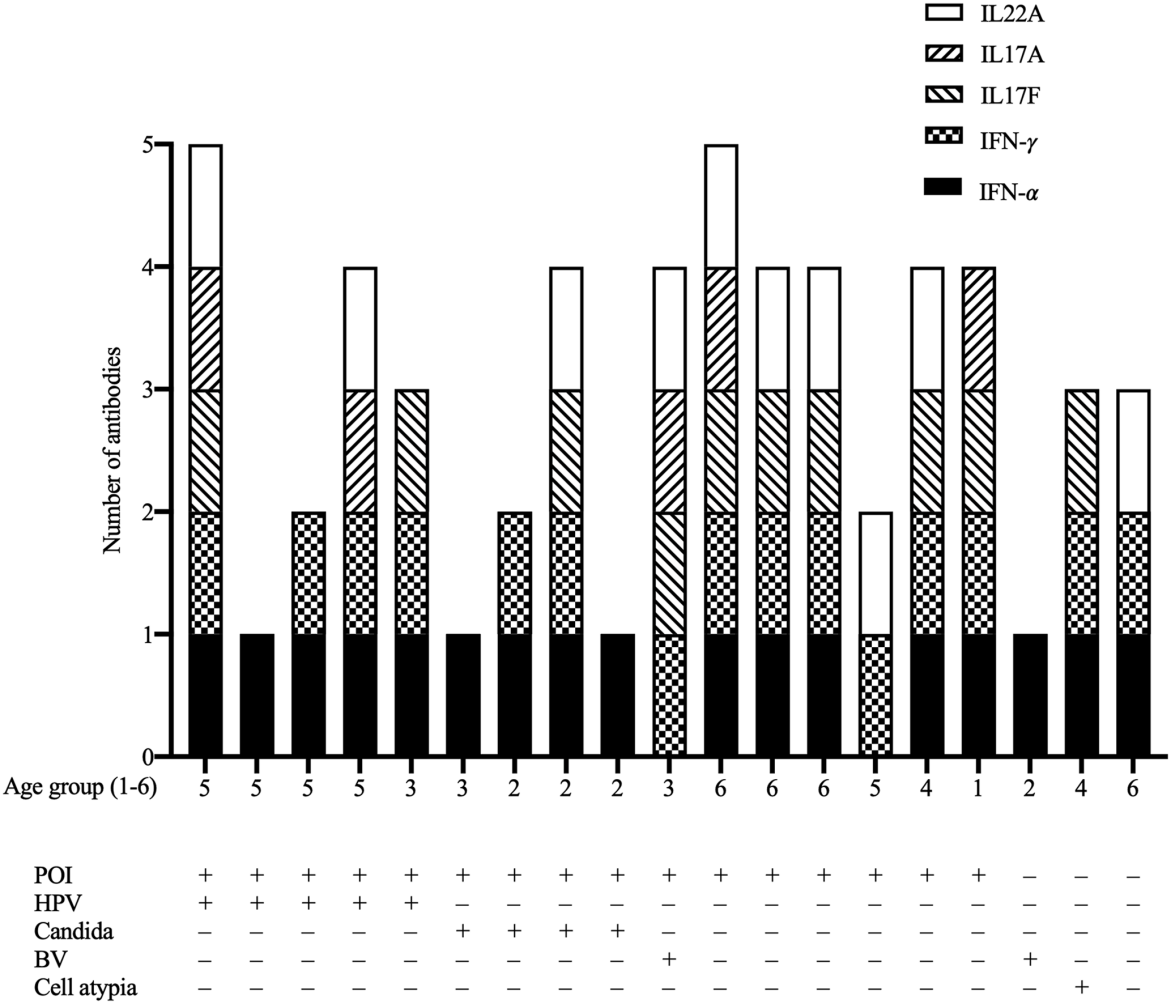
The number of positive anti-cytokine autoantibodies (IFN-α, IFN-γ, IL17A, IL17F, IL22) in the 19 female patients with APECED in relation to age group, premature ovarian insufficiency (POI), human papillomavirus infection (HPV), candidiasis (CMC), bacterial vaginosis (BV), and cervical cell atypia. Age groups: 1, <20; 2 20–29; 3, 30–39; 4, 40–49; 5, 50–59; 6, 60–69 years.

Autoantibodies against IFN-α or IFN-γ were positive in all 19 patients. IL-17A, IL-17F, and IL-22 autoantibodies were found in five, ten, and ten patients, respectively while six (32%) patients had no other cytokine autoantibodies (**Figure 2**). The total amount of positive anti-cytokine autoantibodies did not differ considerably in correlation with age and presence of POI or PAI (*p >*0.05). There was no difference in the prevalence of any measured autoantibody according to the prevalence of any genital infection (*C. albicans*, HPV, bacterial vaginosis; Fisher’s exact test, *p >*0.05).

## Discussion

We carried out a comprehensive evaluation of 19 female patients with APECED to clarify how endocrinopathies and immunodeficiency affect the gynecologic health. In summary, POI was diagnosed in 84% of the patients and 88% of them currently used or had used until physiologic menopause adequate HRT. In most of the women, the morphology and size of the uterus were determined normal for age, menopausal status, and current hormonal therapy. On the contrary to POI, of the 15 patients with PAI, 73% had unmeasurable androgen levels but only three used DHEA substitution. Genital infections were found in almost half of the patients. Genital *C. albicans* infection was diagnosed in four (21%) and HPV infection in five patients (26%). Cervical cell atypia was detected only in one patient. Measured anti-cytokine autoantibodies did not correlate with any of the considered genital infections.

POI was diagnosed in most cases after spontaneous menarche. However, 26% of the patients developed POI early and had primary amenorrhea. AMH values were unmeasurably low in 88% of POI subjects but could be detected in two patients with POI. This represents a typical finding in POI with steroidogenic cell autoimmunity where the pool of growing follicles can initially remain intact (31). Thus, fluctuating ovarian function is typically associated with the early phases of POI and occasional ovulation may happen highlighting the importance of contraception if needed. Early detection of ovarian insufficiency is important to ensure timely pubertal development and adequate growth of uterus with sufficient HRT (32). In our series, the morphology and size of the uterus were determined normal in 76% of women. In case of dyspareunia or genitourinary symptoms, vulvovaginal atrophy should be actively treated by local estrogens (33).

PAI is one of the three classic manifestations of APECED. In our cohort, 15 women had PAI and all also suffered from POI. All androgens were below the detection limit in 73% of the patients with PAI. Because of abnormal hormonal function of both the adrenal cortex and the ovaries, female patients with PAI and POI do not have any normal production of androgens. In our cohort, none of the patients currently used testosterone substitution and only four patients had ever used testosterone, three of them for induction of pubarche. Small case series have shown long-acting testosterone to be effective in the induction of pubarche (34). The only evidence-based indication for testosterone therapy in women is for the hypoactive sexual desire disorder. Effects of testosterone replacement therapy on musculoskeletal health, overall wellbeing and cognition are not clear (34, 35). Only three of the 15 patients with PAI used DHEA substitution. Reported effects of DHEA substitution in PAI are variable; some cohorts show significant improvements in sexual health and libido (18) while others show no effect (19). In a small series of adolescent girls with PAI, DHEA was beneficial to induce pubarche and to relieve psychological distress (36). However, current PAI guidelines do not recommend DHEA substitution automatically for every patient with PAI (37). Regarding patients with POI, the effect of DHEA substitution in the improvement of fertility and successful pregnancies is controversial (38). Nevertheless, since POI often presents with PAI in patients with APECED, DHEA replacement trial could be beneficial in patients with low androgen levels due to both POI and PAI. DHEA substitution in females with PAI may also have positive effects on bone density (19). Further studies are needed to evaluate the potential benefits of DHEA replacement in women with APECED.

In APECED, the reported incidence of CMC at the oral mucosa and other parts of the digestive tract varies between 77% and 100% (6). In an American APECED cohort the prevalence of *C. albicans* in the genital tract was 52% (15). In our cohort the prevalence was significantly lower as only 21-27% of the females with APECED had *C. albicans* growth in the genital tract cultures in the present or an earlier study visit. Hypoparathyroidism has also been shown to associate with impaired immune function. Symptoms and clinical findings varied from asymptomatic to ulcerative and infectious mucosa highlighting the importance of microbiological diagnosis. One of the patients had currently *C. albicans* strain resistant to two tested azoles. Even though the current prevalence of *C. albicans* infections in the genital tract was not high, drug resistance might appear and should be evaluated especially in chronic and recurrent cases.

*C. albicans* is often seen as a commensal microbe at the epithelial surfaces of a healthy individual but can become pathogenic when immunity is compromised (39). Protection against *C. albicans* is thought to be mediated by T cells, particularly by IL-17–producing Th17 cells (40). Th17 cells also produce other interleukins, such as IL-22, that regulate antimicrobial genes and maintain barrier integrity at the epithelial surfaces (41). In patients with APECED, antibodies against IL-17A and IL-17B have been shown to correlate with the development of oral CMC (7, 8). We did not find any correlation between genital tract CMC or other genital infections and the appearance of anti-cytokine autoantibodies. A very recent study suggested that susceptibility to oral CMC is not due to impaired IL-17 immunity in APECED but rather the infiltration of malfunctioning CD4+ and CD8+ T cells in the oral mucosa. These pathogenic T cells cause an excessive IFN-γ/STAT1-response that promotes oral epithelial defects and receptivity for CMC (42). It is not known if a similar autoimmune process is present in other mucosal surfaces in APECED.

In addition to CMC, hormonal deficiencies and defects in mucosal barrier may increase the risk for other infections, as genital infections were found in almost half of our patients. HPV was found in five patients (26%) at the median age of 52 years, and three of them (60%) had high-risk subtypes. The Finnish series of women attending HPV screening during 2003–2005 showed HPV positivity to vary between 2.8–24.2% depending on the age group and prevalence of high-risk subtypes to be 41.2–65.5%. HPV positivity in 50-year-old female patients was 5.1% (43). Based on this, HPV infection was over five times more common in our cohort of females with APECED than in the average Finnish females. HPV infections are known to be more likely to cause cell atypia when the patient has disturbances in immune defense. Even though mutations in *AIRE* cause changes in the T cell functions, we did not see a particularly high incidence of cell atypia in patients with HPV. However, HPV vaccinations could be considered to diminish the incidence of high-risk HPV infections in patients with APECED.

Our study was limited by the small number of patients. Nevertheless, it is noteworthy that our study is the largest gynecologic evaluation ever performed in patients with APECED and the participation rate (61%) was good. Hormonal defects, mucosal health, and even asymptomatic gynecologic infections may impair sexual wellbeing, but without standardized questionnaires, we were not able to study such effects.

In conclusion, APECED predisposes to sex hormone deficiencies and genital infections. The high prevalence of POI and PAI together with immunodeficiency in patients with APECED warrant careful gynecological follow-ups to ensure adequate HRT and treatment of genital infections. As POI is commonly presented together with PAI, all androgens are often very low in patients with APECED. The effects of DHEA substitution on gynecologic well-being should be further explored. Genital infections in female patients with APECED are common and therefore low threshold of taking microbe samples when symptoms are presented, are warranted. The possibility of azole-resistant *C. albicans* strains should be considered when prescribing medications for genital candidiasis. Further studies are needed to explore the mechanisms leading to increased risk for gynecologic infections.

## Data Availability Statement

The datasets presented in this article are not readily available because restrictions apply to the availability of data generated or analyzed during this study to preserve patient confidentiality. The corresponding author will on request detail the restrictions and any conditions under which access to some data may be provided. Requests to access the datasets should be directed to EH elina.holopainen@hus.fi.

## Ethics Statement

Ethical approval was obtained from the Research Ethics Committee of the Hospital District of Helsinki and Uusimaa. Written informed consent to participate in this study was provided by participant or by the participants’ legal guardian / next of kin.

## Author Contributions

SL, AT, OM, and EH contributed to conception and design of the study. VS, SL, AT, and EH were responsible for clinical study visits and data collection. VS was responsible for statistical analysis and wrote the first draft of the manuscript. SL, AT, OM, and EH contributed to the interpretation of the results. All authors contributed to manuscript revision, read, and approved the submitted version.

## Funding

This study was supported by The Helsinki University Hospital; The Päivikki and Sakari Sohlberg Foundation; The Finnish Foundation for Pediatric Research; The Academy of Finland; The Sigrid Jusélius Foundation; The Folkhälsan Research Foundation; The Novo Nordisk Foundation.

## Conflict of Interest

The authors declare that the research was conducted in the absence of any commercial or financial relationships that could be construed as a potential conflict of interest.

## Publisher’s Note

All claims expressed in this article are solely those of the authors and do not necessarily represent those of their affiliated organizations, or those of the publisher, the editors and the reviewers. Any product that may be evaluated in this article, or claim that may be made by its manufacturer, is not guaranteed or endorsed by the publisher.
